# Safeguarding the bedrock of our healthcare system: recommendations to strengthen healthcare worker health in tertiary healthcare institutions

**DOI:** 10.3389/fpubh.2026.1890465

**Published:** 2026-08-14

**Authors:** Matthew Sim, Victoria Ann Lee, Ken Wah Teo, Huay Ling Tay, Montida Lertnimanoradee, Biauw Chi Ong, Elliot Eu, Chuan De Foo

**Affiliations:** 1 Lee Kong Chian School of Medicine, Nanyang Technological University, Singapore, Singapore; 2 Yong Loo Lin School of Medicine, National University of Singapore, Singapore, Singapore; 3 Division of Population Health and Integrated Care, Singapore General Hospital, Singapore, Singapore; 4 Duke-NUS Medical School, Singapore, Singapore; 5 School of Business, Singapore University of Social Sciences, Singapore, Singapore; 6 Design Programme, Khon Kaen University, Khon Kaen, Thailand; 7 Department of Anaesthesiology, Singapore General Hospital, Singapore, Singapore; 8 Occupational and Environmental Medicine Department, Singapore General Hospital, Singapore, Singapore; 9 NUS Saw Swee Health School of Public Health and National University Health System, Singapore, Singapore

**Keywords:** healthcare workers, occupational health services, workplace health screening, preventive health services, continuity of care, primary health care, workplace health promotion, Healthier SG

## Abstract

Healthcare systems face rising demand from aging populations and chronic disease while the healthcare workforce is itself aging and under strain. Employer-based workplace screening may identify unmet health needs, but its value depends on reliable linkage to continuing care and iterative use if aggregated, de-identified data. Drawing on Singapore General Hospital's 2024 mass health screening programme, a rapid review of relevant policy and literature, and multidisciplinary expert input, this policy brief reconceptualizes employer-based screening as the entry point to an integrated, longitudinal occupational health strategy. Six priorities are proposed in this policy brief: integration with national preventive and primary care pathways; evidence-based and equitably financed screening and follow-up; data-informed workforce planning; worker-centered design; preventive oral health; and health promotion beyond screening. We also highlight transferable principles beyond the Singaporean context that may be applied to other health systems.

## 1 Introduction

Healthcare systems worldwide face two converging challenges: escalating healthcare demand driven by population aging and the growing burden of chronic disease, alongside an aging and increasingly constrained healthcare workforce expected to meet these rising demands (1, 2). Although existing policy responses have appropriately emphasized workforce recruitment, deployment and retention (1–3), the ability of healthcare systems to cope with the increase in workload depends not only on workforce numbers, but also on whether healthcare workers (HCWs) remain healthy and capable to sustain high-quality, efficient care.

Employer-based health screening can provide a convenient avenue to identify unmet preventive and chronic-care needs. Yet, where hospital-level screening is offered, follow-up for abnormal findings may remain dependent on individual initiative and disconnected from longitudinal care (4–8).

Singapore offers an instructive setting to examine this gap. Its health system ranks among one of the best-performing globally (9), having emerged from the COVID-19 pandemic with one of the lowest case fatality rates worldwide (10, 11). At the same time, Singapore is also one of the fastest-aging societies with a correspondingly rising chronic disease burden (12), making Singapore a useful case study to examine how its national preventive-care, primary-care centralization, and digital-health reforms can be leveraged to connect institutional health screenings more effectively with continuing care within and beyond the traditional hospital setting.

This policy brief draws on cross-sectional data obtained from the 2024 mass health screening (MHS) programme at Singapore General Hospital (SGH), Singapore's largest tertiary hospital. By identifying gaps in existing mechanisms from detection to appropriate community-based follow-up and workplace health promotion, it then proposes policy directions for Singapore. It also distills transferable principles that may also inform tertiary healthcare institutions across comparable health systems to develop integrated, longitudinal occupational health strategies that reinforce existing workforce capabilities.

## 2 The impetus for policy change

SGH employs approximately 11,000 staff. Its biennial, hospital-wide mass health screening (MHS) programme is offered to full-time employees across clinical, allied health, support care, administrative, and ancillary roles (screening exercise details summarized in Supplementary Annex 1). Throughout this article, the term HCW is used in this inclusive institutional sense. The 2024 MHS programme focused on several key domains, including:

- cardiovascular risk factors, including obesity, hypertension, diabetes, and hyperlipidemia;
- lifestyle behaviors, including physical activity, diet, sleep, mood, smoking, and alcohol use;
- shift work patterns and associated health impacts;
- oral health behaviors and utilization of dental services;
- cancer screening uptake—breast, cervical, and colorectal screening; and
- pre-frailty and frailty status

Compared with the previous 2022 MHS, the 2024 MHS suggested a persistent preventive health gap among staff. Cardiometabolic risk predominated, while sleep duration and quality, smoking, dietary habits, and physical activity were suboptimal. Uptake of preventive health services remained limited, with participation in age-appropriate breast, cervical, and colorectal cancer screening low, and many eligible staff overdue or had never been screened. Dental service utilization was similarly limited, indicating a consistent gap in preventive-care engagement. Overall participation in MHS itself has also declined over successive iterations (results summarized in Supplementary Annex 2).

To translate these findings into policy options, the authors first reviewed the MHS findings to identify major preventive health gaps among HCWs, then undertook a rapid, targeted review of: (i) hospital-level policy documents; (ii) technical reports; (iii) national policy documents via publicly available academic literature; and (iv) relevant gray literature. The resulting healthcare gaps, barriers, and policy opportunities were iteratively grouped into themes and subthemes, which were subsequently refined through consultation with occupational-health leaders in Singapore. Ethics approval for the use of programme data was obtained from the National University of Singapore Institutional Review Board (NUS-IRB-2025-795).

**Figure 1:**
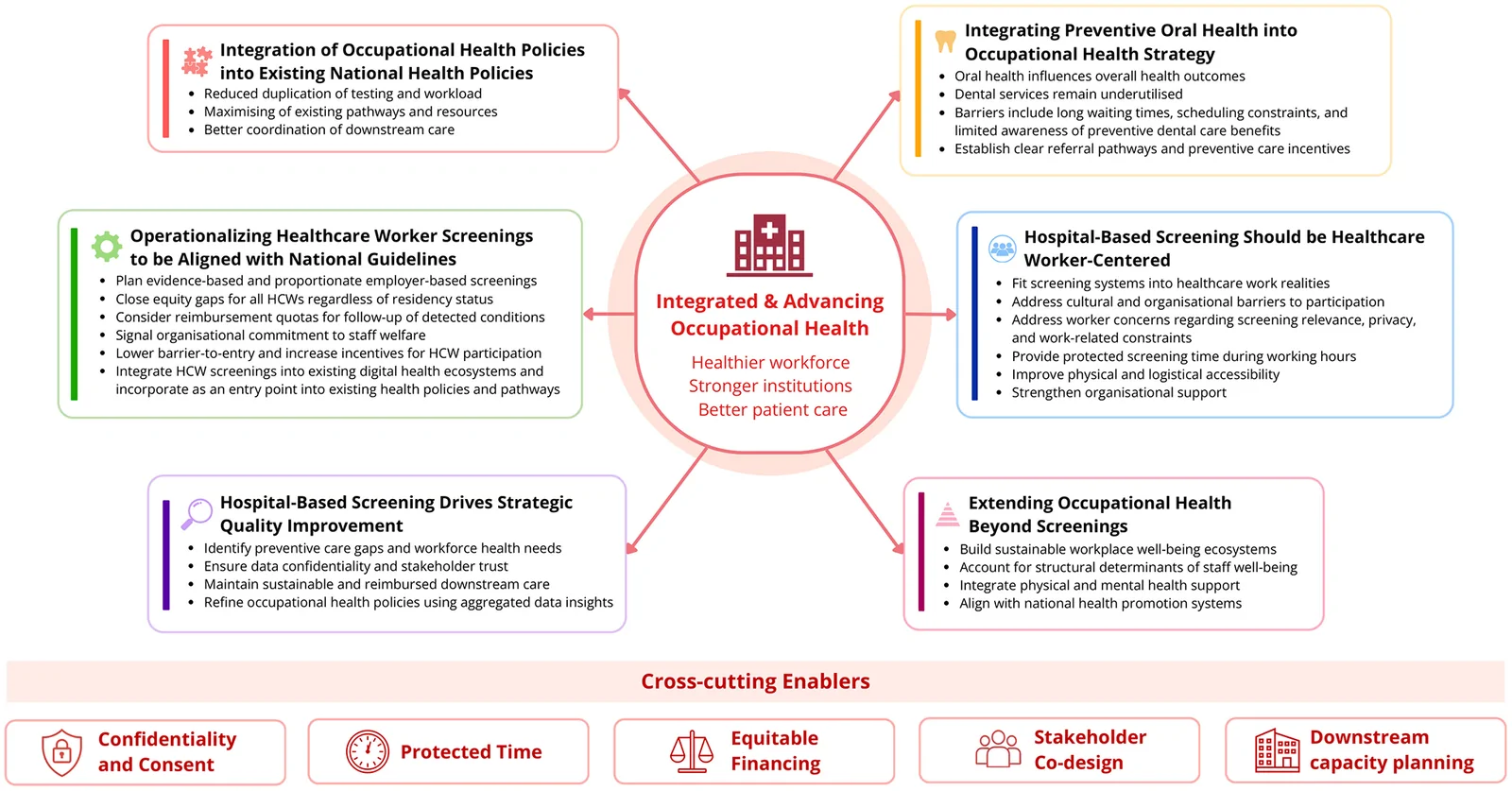
Policy domains for strengthening healthcare worker health in tertiary healthcare institutions. Six interdependent domains derived from the Singapore General Hospital screening findings and policy review: integration with national or equivalent preventive-care pathways; evidence-based and equitably financed screening and follow-up; use of aggregated, de-identified data for workforce-health planning; preventive oral health; worker-centered programme design; and health promotion beyond screening. Together, these domains position screening within a longitudinal occupational-health strategy in support of a healthier workforce, stronger healthcare institutions and improved patient care.

This process identified six interdependent policy domains (Figure 1). The following sections are organized around these domains, with each section first identifying the key policy gaps contextualized to the current policy environment before presenting corresponding recommendations directly addressing the challenges discussed.

## 3 Integration of occupational health policies with existing national health policies

The central policy gap identified by the SGH MHS was the limited integration of HCW screening programmes across Singapore with longitudinal care. Although participants receive results and follow-up advice, subsequent referrals and care generally depend on individual initiative, arranged ad hoc and subject to prevailing charges. This fragmented pathway risks loss to follow-up, duplicated investigations, and diminished engagement (13, 14).

Healthier SG (HSG), a landmark national health system transformation policy introduced in Singapore July 2023 (15), places primary care and community partners at the center of preventive health. Singapore Citizens (SCs) and Permanent Residents (PRs) aged 40 years and above may enroll with a chosen primary care clinic, co-create a health plan, and attend subsequent annual check-ins (16).

HSG Screening (formerly known as Screen for Life), provides nationally subsidized age-, sex-, and risk-appropriate screening from age 18 for eligible groups. There creates opportunity for MHS to build on the existing preventive-care ecosystem, coordinating individuals, screening results, risk profiles (based on lifestyle factors and occupational risks), and care capacity across hospitals, screening vendors, and downstream primary care, dental, and community providers, supported by Singapore's digital health infrastructure.

**Figure 2:**
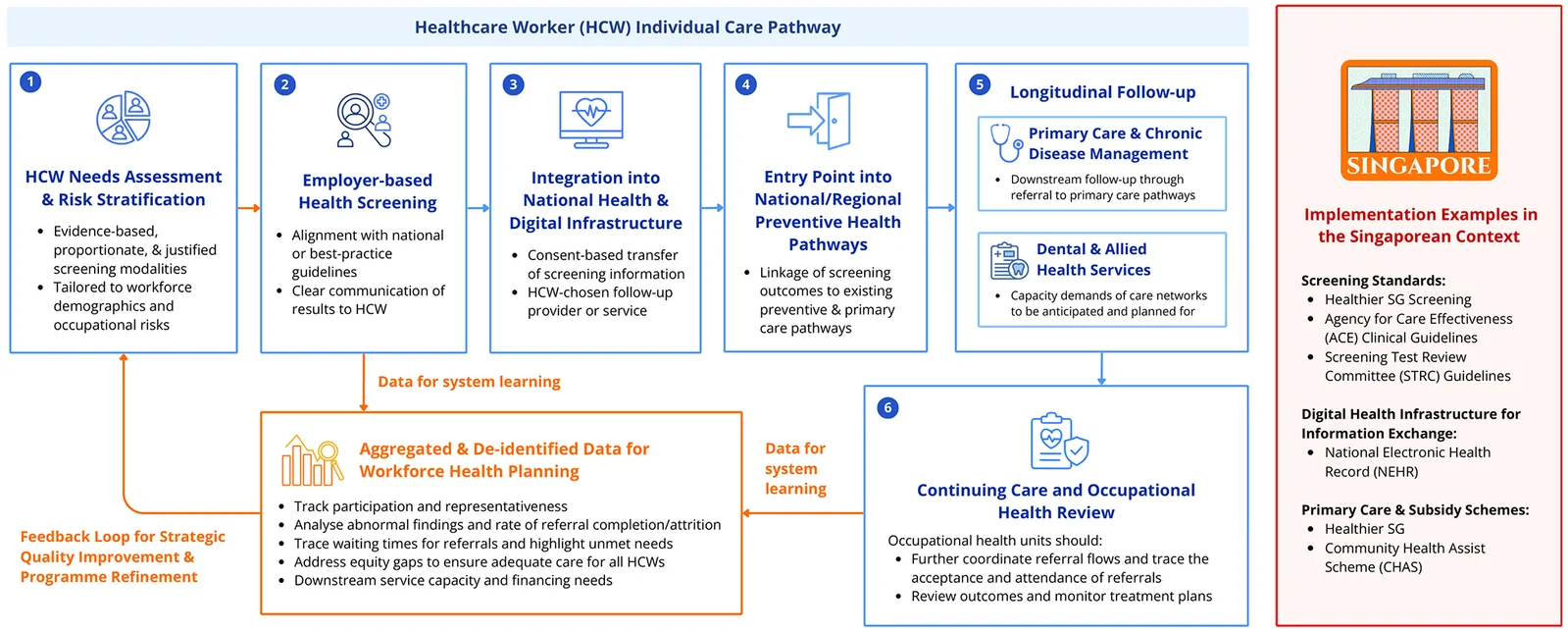
Proposed closed-loop pathway from screening to continuing follow-up. The institutional pathway begins with a healthcare worker needs assessment and risk stratification, followed by evidence-based screening aligned with national or best-practice recommendations and clear communication of results. Screening may serve as an entry point into a national preventive-care pathway where available, or into an equivalent locally arranged pathway. With the worker's consent, relevant results are transferred securely via appropriate digital health infrastructure where available to support longitudinal primary care, dental, or specialist follow-up. Aggregated, de-identified programme data may inform non-punitive workforce health planning and iterative improvement of institutional occupational health policies. The Singapore-specific pathway is presented as an illustrative implementation of these broader, transferable functions.

The following sections outline policy directions for how a tertiary hospital's MHS programme can improve the health of its workers, with HSG providing the Singapore-specific route to community-based follow-up and longitudinal preventive care (17). The pathways enabling collaborative, whole-of-system approach to managing HCW health are depicted in Figure 2, providing easy visualization of on-the-ground processes that facilitate the movement of HCWs and delivery of services across the care pathway.

## 4 Healthcare worker screenings to complement national policies

Participation in the SGH MHS has declined from 61.0% in 2019 to 47.2% in 2022 and 30.9% in 2024, underscoring the need to pinpoint the key areas that perpetuate the decline in numbers. Furthermore, participation also varied across staff groups and age bands, ranging from 43.6% in administration to only 16.3% among medical staff in 2024; similarly, while 34.3% of staff aged 30–39 years participated, this fell to only 14.5% among those aged 70 and above (internal SGH programme data, unpublished). Although the underlying reasons could not be directly ascertained given that these data were collected for descriptive internal hospital-based quality assurance, the observed pattern is consistent with barriers reported in the broader workplace health screening literature where perceived irrelevance, privacy concerns, and poor fit with workers' needs recur as reasons for non-participation (18, 19).

### 4.1 The value-gap in current employer-based screening

Employer-based screening should be evidence-based and proportionate, with a favorable balance of benefit, harm, and cost for the hospital to appreciate the operational value in such initiatives (20). In Singapore, this requires alignment with national guidelines, including HSG Screening (21), Agency for Care Effectiveness clinical guidelines and recommendations from the Screening Test Review Committee (22, 23). Tests supported by robust evidence of clinical and cost effectiveness are suitable for population-level screening, while additional investigations should be reserved for higher-risk individuals following clinical assessment, including occupational predispositions (23).

Under HSG Screening, SCs and PRs can already access age-, sex-, and risk-appropriate cardiovascular, cervical and colorectal screening on a heavily subsidized basis, with one post-screening consultation where required (21); eligible SCs enrolled under HSG receive additional subsidies for screening tests and vaccinations at their enrolled clinic (21). Government funded portable subsidies such as Community Health Assist Scheme (CHAS) and HSG Chronic Tier subsidies then support selected chronic disease management in primary care, such as the coverage of chronic medication costs (24, 25). In light of this, HCWs may reasonably prefer to screen through their regular primary care physician if employer-based screening offers no clear advantage or risks fragmented follow-up or further out-of-pocket costs.

### 4.2 Equitable financing across the care continuum

Employer-based screening must therefore offer value beyond a free test alone, etched in the principle of accessible healthcare for all HCWs, with the mindset that all HCWs play a contributory role in the health system. Hence, MHS provision should be tiered and equity-based to complement existing national subsidies and take into consideration the differences in coverage across HCWs of different residency statuses (21). This ensures that health services are not out of reach for any HCW population.

While most (if not all) tertiary healthcare institutions in Singapore already provide employee outpatient benefits, employers should consider offering additional ring-fenced support (i.e. funding reserved exclusively for a defined purpose and non-divertible to other budget lines) for chronic conditions detected through staff screening, such as a capped reimbursement (per employee) for follow-ups at referred community providers or outpatient clinics. The design and scale of such support should importantly be guided by utilization patterns and equity considerations, among others. Such benefits also signal organizational commitment to staff welfare, and may strengthen morale, improve retention, and make the benefits of screening more tangible to workers.

### 4.3 Integrating with national/regional preventive care pathways

New policies need to lower the barrier for HCWs to take up integrated MHS-primary care journeys.

With hospital-to-community initiatives already a successful model of care for patients with chronic diseases (26, 27), employer-based screening can serve as either a convenient entry point into, or a regular checkpoint within an existing primary care relationship.

For the SGH MHS, HCWs eligible for HSG but not yet enrolled could be offered assisted enrolment and invited to nominate their preferred follow-up site. Those already enrolled should receive a clear plan for review with their regular clinician. This way, the employer functions as a convenient screening and referral node within a broader longitudinal pathway, with chronic disease care remaining anchored in primary care and community follow-up, consistent with HSG's policy intent. Primary care physicians can also provide additional health guidance tailored to HCWs within their workplace environments, helping to optimize overall wellbeing, particularly given that work occupies a substantial portion of an HCW's daily life.

Operationally, screening results should flow into existing digital ecosystems and, with the worker's consent, be made available to the worker's regular primary-care clinician. This is supported by the phased implementation of the Health Information Act since January 2026, requiring licensed healthcare providers to contribute selected health information to the National Electronic Health Record (NEHR) over time (28). As electronic medical record platforms are also gradually enabled with HSG and NEHR connectivity, authorized clinicians across care settings will be able to review investigations performed during mass screenings, allowing them to recommend appropriate, non-duplicative services and strengthen the role of staff health screening as a key component of preventive care.

## 5 Hospital-based screening drives strategic quality improvement

### 5.1 Aggregated data for workforce health planning

Given that tertiary healthcare clusters in Singapore are each outfitted with occupational health units, aggregated and de-identified HCW screening data allows for upstream research into recurrent preventive care gaps and broader health needs. This further facilitates data-driven and needs-based interventions tailored to the specific health, wellbeing, and occupational risk demographics of HCWs. When workplace-health policies are evidence-informed, palatable, and operationally sound, they are poised in a better position to improve employee health and productivity (29, 30). Beyond occupational health interventions, findings can also be used within a culture of improvement and learning, with continuing medical education for occupationally relevant conditions made available to both in-house and community-based providers with a focus on preventive health management (31–33).

### 5.2 Safeguarding confidentiality and trust

Data use must be strictly governed, and employers must not have access to identifiable individual health information while protecting HCWS' data from targeted third-party vendor attacks or inadvertent staff driven leaks (34). Enforcing and emphasizing this is essential to maintaining stakeholder trust, especially among HCWs as they may be concerned that disclosure of personal health information could affect employability or career progression (35).

### 5.3 Planning for upstream and downstream resource allocation through collaborative system design and data feedback loops

Mass screening can impose downstream demands on community providers and occupational health units, and these effects should be anticipated, monitored and matched with adequate financing and capacity. Therefore, the volume, case-mix and referral destinations should be tracked to direct care to the most appropriate setting. Where additional financing is required, it should align with pre-existing national or regional payment structures and primary care funding models (in Singapore's case, HSG & CHAS) to minimize additional administrative workload and duplicative systems. Approaches to reimbursement should be explored with all stakeholders involved moving forward.

Ultimately, a co-creative approach to MHS programmes and community follow-up will require whole-of-hospital (inclusive of all hospital departments) and whole-of-health-system (inclusive of all health and non-health sectors) efforts. To that end, regular feedback sessions with HCWs, relevant hospital departments, occupational and primary care providers, community partners, and administrators should guide the refinement of screening programmes and community follow-up models to improve feasibility for all parties involved and to address unintended consequences before policy changes are made at-scale.

## 6 Integrating preventive oral health into occupational health strategy

Oral health is closely associated with broader health outcomes, including cardiometabolic diseases (36, 37), but remains a historically neglected part of health screenings at tertiary hospitals writ large (38). Therefore, beyond the present core nationally recommended screening package, employers should consider evidence-based extensions that address unmet needs in the working-age population, particularly the under provision of dental and oral-health services.

Within SGH, findings from the 2024 Health Screening suggest that uptake of preventive dental care, although improved since 2022, remains suboptimal. In 2024, 34.6% of surveyed staff reported seeing a dentist at least six-monthly, broadly mirroring data from the 2019 National Adult Oral Health Survey (NAOHS 2019), in which only 26.2% reported six-monthly attendance (39). This falls short of the recommended six-monthly to annual preventive check-ups (40, 41). In 2022, fewer than 35% of SGH staff utilized their entitled dental benefit, again reflecting broader national patterns of underutilization (38).

### 6.1 Barriers to preventive dental attendance

Substantial perceptual and opportunity-related barriers to dental healthcare utilization among HCWs persist. In NAOHS 2019, the most common reason for irregular dental visits was the perception that there was no need to see a dentist (55.1%) (39). For health institutions, the policy response should therefore be greater integration of dental health into the broader staff health agenda in the same way that workplace campaigns have normalized awareness of smoking cessation, physical activity, and healthier diets. In collaboration with institutional dental departments, preventive oral-health messaging and referral prompts could be incorporated into future screening exercises and follow-up communications.

Beyond awareness, cost-related concerns (38.5%), and lack of time (15.2%) were the next most commonly cited barriers in NAOHS 2019 (39), likely to be especially relevant for HCWs. Long waits for non-emergency dental services in the public sector, including reports in 2024 of waits of up to a year (38), may discourage timely preventive attendance even if more recent data suggest that median waiting time had improved to 3 months in 2025 (42). MOH dental fee benchmarks suggest that routine preventive visits (comprising consultation, polishing, and scaling) may consume a substantial proportion of available staff entitlement even before any restorative treatment is required (43).

### 6.2 Building preventive dental pathways for healthcare workers

There are, at present, a lack of well-established public hospitals down-triaging (meaning to refer to lower levels of care) to private dental clinic processes in Singapore, despite the private sector dominating the dental healthcare space. Employers can address this by first playing a coordinating role in establishing clear cross-referral pathways to community private dental clinics or public dental services in advance while creating dedicated preventive appointment capacity. Furthermore, schemes that specifically incentivize preventive attendance via increased financial support for routine preventive dental care may be explored in depth. This includes enhanced reimbursement for preventive-only visits (potentially dovetailed with institutions' health screenings), as well as allocated time for staff to attend dental appointments, thereby smoothing uptake across the year and reinforcing preventive norms.

Such network-based approaches are consistent with broader health system objectives and can help stabilize demand while improving predictability for dental providers (14, 44). Furthermore, evidence from the United States and Japan suggests that workplace oral health initiatives may be associated with lower dental and medical expenditure, reduced absenteeism and presenteeism, with potentially favorable cost-benefit to employers (45–48).

## 7 Hospital-based screening should be healthcare worker-centered

Health related activities, including health screenings, should place HCWs at the center of its operational decisions, in terms of resource provision and meeting high-quality standards that serve the recipients the most optimally. The World Health Organization notes that safeguarding workers' health improves productivity, job satisfaction, and retention, while reducing the financial burden associated with illness, injury, and absenteeism (49). All HCWs should therefore have equitable access to preventive health services without undue financial or operational barriers.

At the individual level, key challenges include health literacy and perceived relevance. Not all HCWs may appreciate the importance of regular health screening or be aware of their employee health benefits. More broadly, employee-facing studies have also identified perceived irrelevance, privacy concerns, and poor fit with workers' needs and preferences as barriers to screening uptake (18, 19). There may remain a perception that leaving work for screening or health-related appointments increases workload on fellow colleagues, thus impacting workplace relationships. Addressing this mindset will require a broader cultural shift, in which preventive health attendance is normalized as part of a responsible workforce.

### 7.1 Normalizing preventive health screenings in workplace culture

Policies should be designed to minimize structural constraints to HCWs' participation in preventive health screening. Employee health screenings should reduce the practical costs of attendance by taking place during working hours with no impact on employees' rest days. For HCWs with high-intensity or shift-based environments, protected screening time, on- or near-site access, and clear departmental arrangements for staff release should be enabled, such that all workers get equal time rostered for preventive and occupational health attendances.

Employers should also move beyond generic posters or email reminders. Screening should be embedded in staff onboarding as an explicit employee benefit, accompanied by clear information on eligibility, confidentiality, follow-up arrangements, and available financial support. Visible participation by senior clinicians and administrators, as well as having hospital departments facilitating attendance rather than merely permitting it if workload allows will further signal the importance of health screening.

## 8 Extending occupational health beyond screenings

Health systems must be structured to foster workplace environments that support healthier lifestyles and overall wellbeing among HCWs as a foundational step toward strengthening healthcare workforce resilience and improving population health. Singapore's aging population, compounded by an aging healthcare workforce and an increasing retirement age, have intensified healthcare demands and may predispose HCWs to chronic conditions and prolonged working hours amid rising patient loads (50). Burnout among healthcare professionals is increasingly recognized as a critical concern, characterized by emotional exhaustion, reduced empathy, and detachment from work, ultimately affecting quality of patient care, workforce sustainability, and predisposing HCWs to poorer health outcomes (51).

### 8.1 Redesigning work to support health and wellbeing

As the number of patients at tertiary hospitals go on an uptrend, burdens placed on HCWs will inevitably increase. Higher levels of work-related stress are invariably associated with poorer health in HCWs (52). To mitigate workforce strain, Singapore has expanded capacity through additional tertiary hospitals and recruitment of HCWs (53). However, unbridled capacity building alone is unlikely to be sufficient or financially sustainable. Strategic investment in technology, particularly artificial intelligence (AI), offers opportunities to streamline repetitive clinical and administrative tasks, thereby alleviating cognitive load. Concomitantly, AI-enabled systems can support functions such as consolidation of investigation results, documentation, and administrative workflows, improving productivity in both clinical and non-clinical settings (54).

Beyond structural reforms, fostering resilience among HCWs requires attention to work-life balance, adequate rest, and opportunities for physical activity. In this broader context, hospital-based health screening should be seen as one component of a wider workforce health tapestry. Screening can identify unmet health needs and facilitate linkage to follow-up care, particularly through team-based models involving occupational health physicians, family doctors, relevant specialists and ancillary providers to ensure continuity of care beyond the workplace. However, it should run parallel to measures that address the structural determinants of staff wellbeing, including workload optimization, reduction of excessive working hours, and improved access to exercise opportunities and healthier food options within the workplace.

### 8.2 Reframing hospitals as health-promoting workplaces

HCWs should be encouraged to view hospitals as more than sites of occupational commitments but environments that champion the health and wellbeing of their own workforce. Even within the limited hospital grounds, selected areas can be purposefully repurposed into rest areas, exercise hubs, sleeping pods, or social spaces that foster social and physical engagements beyond the usual routines of daily work. Fitness classes, gyms and counseling outposts can provide a welcome respite amid a demanding day.

### 8.3 Supporting healthier behaviors through workplace benefits and national platforms

Integrating workplace health promotion into organizational policy while leveraging existing national health policies may produce synergistic effects that strengthen implementation and optimize resource utilization. Work-related benefits such as exercise class passes, healthy cooking classes, or gym memberships may also make healthier behaviors easier to sustain (55–57). These initiatives offer practical avenues for health improvement whilst strengthening morale, camaraderie and institutional culture. In doing so, they may help to mitigate mental health stressors and occupational concerns less likely to be addressed through generic, non-healthcare-worker-led wellness activities. Given evidence of positive economic outcomes and employer benefits (58–60), this area of policy refreshment should be explored.

Such wellbeing activities can also leverage existing platforms for activity tracking, event registration, team-based challenges, reminders, and incentives. In Singapore, the Healthy 365 application, a free government-initiated mobile health application widely used by Singaporeans to track community-based health activities, could similarly be extended to workplace events, allowing HCWs to earn health points and redeem prizes both at and beyond work (61). Inter-departmental step-count or sleep-hygiene challenges can leverage smart watches provided by the Health Promotion Board to all eligible Singaporeans, improving engagement with physical activity and broader health-promoting behaviors through gamification (62).

## 9 Beyond Singapore: applying the findings across health systems

The gaps raised in this policy brief are not unique to Singapore, instead being present in most health systems and tertiary hospitals (63). The lack of assimilation of tertiary level, employer-based screening initiatives with continuing care, underutilization of dental services and lack of designated time to participate in screening events among other factors are well-known gaps globally (64). Safeguarding the health of HCWs should therefore be a topmost priority, itself an essential component of workforce sustainability and health-system resilience.

Singapore illustrates how several enabling mechanisms, including HSG and HSG Screening, Healthier SG, NEHR systems and subsidies like CHAS and HSG Chronic Tier, facilitate the integration of institutional health screening with primary care, information exchange, and affordable follow-up. However, not every health system has the political will or enabling governance to reorganize care around a strong primary care foundation and facilitate seamless patient movements across healthcare interfaces. Similarly, nationwide interoperable health information systems and portable healthcare financing schemes remain beyond the immediate reach of many countries.

Despite these challenges, such prospective transformations provide a clear strategic direction for strengthening screening care models and improving care coordination. Even for countries where primary care is established but records are not interoperable, consent-based transfer of screening to the HCW's chosen provider can achieve similar continuity even without full digital integration. Overall, tertiary institutions still stand much to gain by employing cross-cutting enablers such as preserving confidentiality and consent, carving out protected time for employees, supporting equitable financing, engaging in co-design with relevant stake holders, and prospectively planning downstream capacity.

Ultimately, ensuring that occupational health interventions do not operate in silos and are able to initiate reliable follow up care, while providing adequate and holistic support to both HCWs and downstream providers, would make significant strides toward advancing equitable access to high-quality preventive and continuing care for HCWs while strengthening workforce and health-system resilience.
