# Correction: Safeguarding the bedrock of our healthcare system: recommendations to strengthen healthcare worker health in tertiary healthcare institutions

**DOI:** 10.3389/fpubh.2026.1970279

**Published:** 2026-09-01

**Authors:** Frontiers Production Office

**Affiliations:** Frontiers Media SA, Lausanne, Switzerland

**Keywords:** healthcare workers, occupational health services, workplace health screening, preventive health services, continuity of care, primary health care, workplace health promotion, Healthier SG

The **Figures** were in the wrong order. The images of **Figures 1**, **2** were reversed. The order has now been corrected.

The original version of this article has been updated.

