# Supplementary material for: Safeguarding the bedrock of our healthcare system: recommendations to strengthen healthcare worker health in tertiary healthcare institutions
Source: Front Public Health. 2026 Aug 14;14:1890465. doi: 10.3389/fpubh.2026.1890465 (PMC13522206; doi:10.3389/fpubh.2026.1890465)
Supplement: Supplementary file 1 [file Supplementary_File_1.docx]

Supplementary Material

# Annex 1: Singapore General Hospital’s Mass Health Screening Exercise

All Singapore General Hospital (SGH) staff were invited to participate in the Mass Health Screening Exercise 2024, a hospital-wide staff health screening programme typically held biennially since 2019. The 2024 exercise was held over 10 days in August 2024 on hospital premises by SGH’s occupational health unit, in collaboration with an external private primary and preventive healthcare provider, and was free for all participants.

Staff were informed prior to taking part that their biomedical and survey data would be used in aggregate for hospital quality improvement purposes. Ethics approval for the use of data for the purposes of research was obtained from the National University of Singapore’s Institutional Review Board (NUS-IRB-2025-795). There were no exclusion criteria as long as staff were employed by SGH at the time of screening. Participation was voluntary, and staff could decline or stop participating at any point with no penalty.

The data collection exercise covered the following:

A. Personal Particulars
B. Physical Activities
C. Chronic Disease History
D. Cigarette Smoking
E. Alcohol Consumption
F. Cancer Screening Programmes
G. Mental Health
H. Oral Health
I. Dietary Practices and Nutrition
J. Sleeping Habits

The biomedical investigations included:

A. Biometric Measurements - Blood Pressure, Body Mass Index
B. Glycaemic assessment - Glycated Haemoglobin A1C, Random Blood Glucose
C. Lipid Profile - Total Cholesterol, Low-Density Lipoprotein, High-Density Lipoprotein, Triglycerides
D. Faecal Occult Blood Test (for staff aged 50 & above)
E. Frailty Screening (for staff aged 50 and above)

Optional Test (self-funded):

- 3 in 1 package (Full Blood Count, Kidney Function Test & Liver Function Test)

All data were de-identified and analysed only in aggregate so data could not be traced back to any specific member of staff. The dataset was kept in a password-protected Excel file, with access restricted to research team members. For the purposes of this policy brief, specific datasets are not featured but broad data trends are reported in the main manuscript to provide context to the gaps identified and support the recommendations set forth.
