# Supplementary material for: Safeguarding the bedrock of our healthcare system: recommendations to strengthen healthcare worker health in tertiary healthcare institutions
Source: Front Public Health. 2026 Aug 14;14:1890465. doi: 10.3389/fpubh.2026.1890465 (PMC13522206; doi:10.3389/fpubh.2026.1890465)
Supplement: Supplementary file 2 [file Supplementary_File_2.docx]

Supplementary Material

# Annex 2: Findings from Singapore General Hospital’s Mass Health Screening

Overall participation in Singapore General Hospital’s (SGH) Mass Health Screening (MHS) programme declined substantially over time, from 61% in 2019 to 47.2% in 2022 and 30.9% in 2024. Participation also varied greatly by staff groups and age bands in 2024. Medical staff had the lowest participation rate (16.3%), followed by ancillary staff (20.3%), nursing staff (29.3%), allied health, pharmacy and research staff (40.1%), and administrative staff (43.6%). Similarly, while 34.3% of staff aged 30–39 years participated, this fell to 14.5% among those aged 70 and above, highlighting the need for more targeted engagement strategies.

1. Cardiometabolic Health and Lifestyle Indicators

Among MHS participants, some cardiometabolic indicators showed improvements from 2022 to 2024. The gender-adjusted diabetes^[1]^ prevalence decreased from 3.6% to 2.0% (-1.6%). Hypertension^[2]^ also decreased slightly, from 18.6% to 18.4% (-0.2%). Similarly, the gender adjusted prevalence of high low-density lipoprotein cholesterol levels^[3]^ (LDL-C) decreased from 15.1% to 13.7% (-1.4%), with crude prevalence declining from 13.4% to 11.9% (-1.5%).

Despite these improvements, rising obesity and suboptimal lifestyle behaviours remain pressing concerns. From 2022 to 2024, median body mass index (BMI) rose from 23.2 to 23.6, remaining within the overweight range for Asians, with gender-adjusted obesity prevalence rising from 12.1% to 13.3% (+1.2%), and crude prevalence increasing from 12.2% to 13.5% (+1.3%). In 2024, 60% of participants reported exercising one day or fewer per week, while 23.3% of able participants reported never using the stairs. Smoking prevalence also saw a small increase from 1.9% to 2.1% (+0.2%).

2. Preventive Health Uptake

Preventive health uptake, particularly age-appropriate cancer screening and regular dental attendance, remained low and represents an important area for intervention. In 2024, up to 60.3% of eligible participating female staff had not undergone mammography within the preceding two years. Similarly, at least 58.1% of staff lacked adequate cervical cancer screening^[4]^.

Colorectal cancer screening uptake was also low, with only 17.7% having completed a faecal occult blood test (FOBT) in the preceding year and 52.7% reporting never having undergone FOBT. However, these figures may underestimate true screening rates as colonoscopy and age eligibility were not accounted for. Additionally, 43.1% of staff did not opt in for FOBT screening, although their reasons were not recorded.

In 2024, 34.6% of participants reported attending a dentist at least once every six months, compared with 20.8% in 2022. A further 36.1% reported attending at least once annually. Separately, only 34.88% of staff utilized their dental benefit in 2022 (data from 2023 onwards are not yet available).

1. Defined as either a relevant past medical history or glycated haemoglobin (HbA1c) ≥7.0%
2. Defined as systolic blood pressure ≥140 mmHg, diastolic blood pressure ≥90 mmHg, or a relevant past medical history of hypertension.
3. Defined as LDL-C ≥4.1 mmol/L
4. Defined as the absence of a Papanicolaou smear or human papillomavirus test within the preceding five years.
